# MPH-M, AODV-M and DSR-M Performance Evaluation under Jamming Attacks

**DOI:** 10.3390/s17071573

**Published:** 2017-07-05

**Authors:** Carolina Del-Valle-Soto, Carlos Mex-Perera, Raul Monroy, Juan A. Nolazco-Flores

**Affiliations:** 1 Universidad Panamericana. Facultad de Ingeniería. Prolongación Calzada Circunvalación Poniente 49, Zapopan, Jalisco 45010, Mexico; 2 Telemática Telemetría y Radiofrecuencia, Francia 1717, Col. Moderna, Guadalajara 44190, Mexico; carlosmex@ttr.com.mx; 3 Escuela de Ingeniería y Ciencias, Tecnologico de Monterrey, Campus Estado de México, Carretera al lago de Guadalupe Km 3.5, Col. Margarita M. de Juárez, Atizapán 52926, Mexico; raulm@itesm.mx; 4 Department of Electrical and Computer Engineering, Tecnológico de Monterrey, Ave. Eugenio Garza Sada #2501 Sur, Monterrey 64849, Mexico; jnolazco@itesm.mx

**Keywords:** Wireless Sensor Networks, jamming, routing algorithms

## Abstract

In this work, we present the design of a mitigation scheme for jamming attacks integrated to the routing protocols MPH, AODV, and DSR. The resulting protocols are named MPH-M (Multi-Parent Hierarchical - Modified), AODV-M (Ad hoc On Demand Distance Vector - Modified), and DSR-M (Dynamic Source Routing - Modified). For the mitigation algorithm, if the detection algorithm running locally in each node produces a positive result then the node is isolated; second, the routing protocol adapts their paths avoiding the isolated nodes. We evaluated how jamming attacks affect different metrics for all these modified protocols. The metrics we employ to detect jamming attack are number of packet retransmissions, number of CSMA/CA (Carrier Sense Multiple Access with Collision Avoidance) retries while waiting for an idle channel and the energy wasted by the node. The metrics to evaluate the performance of the modified routing protocols are the throughput and resilience of the system and the energy used by the nodes. We evaluated all the modified protocols when the attacker position was set near, middle and far of the collector node. The results of our evaluation show that performance for MPH-M is much better than AODV-M and DSR-M. For example, the node energy for MPH-M is 138.13% better than AODV-M and 126.07% better than DSR-M. Moreover, we also find that MPH-M benefits much more of the mitigation scheme than AODV-M and DSR-M. For example, the node energy consumption is 34.61% lower for MPH-M and only 3.92% and 3.42% for AODV-M and DSR-M, respectively. On throughput, the MPH protocol presents a packet reception efficiency at the collector node of 16.4% on to AODV and DSR when there is no mitigation mechanism. Moreover, MPH-M has an efficiency greater than 7.7% with respect to AODV-M and DSR-M when there is a mitigation scheme. In addition, we have that with the mitigation mechanism AODV-M and DSR-M do not present noticeable modification. However, MPH-M improves its efficiency by 8.4%. We also measure the resilience of these algorithms from the average packet re-transmissions perspective, and we find that MPH-M has around a 15% lower change rate than AODV-M and DSR-M. The MPH-M recovery time is 5 s faster than AODV-M and 2 s faster than DSR-M.

## 1. Introduction

Wireless Sensor Networks (WSNs) [1] are a set of interconnected electronic devices with small processing and (wireless) communication capabilities. For many applications, it is desirable that wireless sensor devices remain fully operational for a long period. For example, a WSN can be designed to transmit prominent information about a forest, such as a temperature or humidity. Frontier research in WSNs hence involves the development of mechanisms for efficient energy management, enabling sensors to work for a longer time without replacing the battery.

Recent advances in both computing and communications have caused a significant shift in research on sensor networks, i.e., the use of cheap and tiny sensors based on micro-electromechanical systems technology, connected via wireless communication, and making inexpensive low-power processors. This allows for the deployment of ad hoc wireless networks suitable for many applications, including environmental monitoring, security monitoring or object tracking.

There are applications where sensitive information is transferred through the WSN, i.e., cryptographic key exchange. Typical threats may be communications eavesdropping, nodes impersonation, data corruption, etc. A first countermeasure could involve the use of cryptography mechanisms, i.e., the IEEE 802.15.4 standard describes a series of cryptography mechanisms that guarantee a set of properties implying secure communication [2].

However, the implementation of cryptographic algorithms cannot reduce the risk due to other threats in wireless communications; for example, intentional interference, called jamming, where the receivers are saturated with spurious signals avoiding the reception of legitimate data. Usual effects of jamming are higher energy consumption when the nodes increase the re-transmissions rate leading to a lower performance of the network.

We propose in this work a mitigation mechanism for reducing the effects of jamming attacks in WSNs, we have modified MPH (Multi-Parent Hierarchical) [3], AODV (Ad hoc On Demand Distance Vector) [4] and DSR (Dynamic Source Routing) [5], the new versions of the protocols that include the mitigation scheme are called MPH-M, AODV-M and DSR-M.

Detection of jamming attacks is performed with an anomaly model, which considers the following variables: packet re-transmissions rate, number of CSMA/CA (Carrier Sense Multiple Access with Collision Avoidance) retries waiting for an idle channel, and energy consumption. After a positive detection is done, a reactive phase starts, where the self-configuring capabilities of the routing protocols are triggered for avoiding the jamming area and reducing the impact on the whole network.

### Objective

The purpose of this paper is to minimize the effects of jamming attacks by adding a mitigation mechanism to well-known routing protocols, which we called MPH-M, AODV-M and DSR-M.

We describe the organization of the rest of the paper. Section 2 introduces the model implemented in this work for the jamming attack. In Section 3, we present related work. Section 4 proposes a scheme for the mitigation of jamming attacks. Section 5 discuss the anomaly model and the behavior of the variables selected for the jamming detection. Section 6 presents the results. Finally, conclusions are given in Section 7.

## 2. Reactive Jamming Attack

In this section, we will explain the jamming attack model, which in general produces a Denial of Service (DoS) effect in the target nodes [6].

A jammer is defined in [7] as “... an entity who is purposefully trying to interfere with the physical transmission and reception of wireless communications”.

Jamming models can be stationary or mobile whether or not the attacker stays in the same position during the attack. The detection of jammer that moves through the network is more difficult, because the detector needs to consider the dynamic behavior of the channels in each node [8].

Jamming models can be also classified as constant or deceptive. A constant jammer continuously sends a radio signal or random bits to the channel without following any MAC-layer protocol; a deceptive jammer constantly injects regular packets to the channel without any gap between subsequent packet transmissions.

In the random jamming model, the jammer switches between active and sleeping modes; when the attacker is in the active mode, it behaves as either a constant or deceptive jammer. In contrast, in the reactive jamming model, the attacker remains quiet while the channel is idle; when it detects a packet, then it sends an interference signal in order to corrupt the ongoing transmission [9].

In reactive jamming, for MAC-layer protocols using Backward Error Correction, such as go-back-n or repetitive select, the attack would be successful if at least one bit of the packet is altered. Reactive jammer is energy efficient, because only transmits when it detects a packet, and therefore it is harder to detect since it could mimic packet collisions that are caused by normal traffic activity in the network [9,10].

In a previous work, we have shown that some performance metrics, such as the number of packet retransmissions, the number of retrials in the inner loop of the CSMA/CA algorithm, and the energy consumption are all affected by a jamming attack [11].

In this work, we will evaluate the performance of a number of WSNs for MPH-M, AODV-M, DSR-M respectively) with an attacker that uses a reactive jammer model.

**Figure 1:**
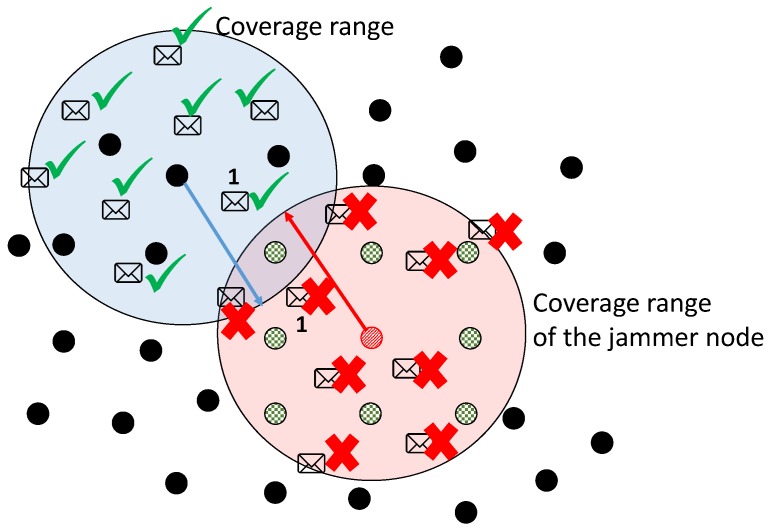
Reactive mechanism for packet collision.

Figure 1 shows how the mechanism of reactive jamming behaves; the shaded area represents the coverage area of a given node. In this example, we depict two coverage radii. One for the jammer node and another for an arbitrary node of the network. We can observe that there are two cases for the resultant behavior due to the mechanism of reactivity. One is when a packet is directed to any node that is within the coverage area of the jammer node. The second case is when a packet is directed to a node that is outside the coverage area of the jammer node. In the latter case, the jammer still tries to make packets collide, but since the receiver is far beyond its coverage area then the packet can correctly reach its destination without interference. This last alternative is shown in the figure by means of the packets marked with the number 1, which is actually the same packet. Nodes marked with an *X* are nodes that are within the coverage area of the jammer node and their packets can be intercepted. Nodes marked with ✓ are nodes that are beyond the coverage area of the jammer node.

## 3. Related Work

There is a number of works related to this research that deserves to be reviewed.

### 3.1. Jamming Detectors

Jamming detectors are relatively easy to design. In this section, we will review some of the techniques proposed by other authors.

In [12], the jamming detector analyzes throughput, energy in the nodes, and end-to-end delay as performance metrics. In Pelechrinis et al. [13], Packet Delivery Ratio (PDR) is used to detect jamming. Moreover, in [14], the author observes collisions rate and SNR to detect the jammer node [14]. Here, authors analyze widely known metrics; however, they overlook that these metrics can be altered by momentary interferences, or sudden changes in the communication channel. The performance metrics we have studied in our work are directly related to routing and traffic and are more specific with respect to changes in the network. In addition, they are linked to topology changes as a result of reconfigurations due to attacks.

In [15], the author discusses two strategies: proactive and reactive channel-hopping in order to understand which of them is more resistant to a jamming attack. To test this hypothesis, they analyze two performance metrics: availability and energy. This work adequately complements ours because the authors contrast the two nature of the protocols in WSNs. It is precisely what we want to point out, that our proposed protocol, MPH, with proactive nature, presents better performance compared to AODV and DSR, two protocols widely studied with reactive nature. This performance is analyzed with more metrics such as: overhead, retransmissions, CSMA retries, energy, delay, and so on.

Strasse et al. [16] propose to detect a proactive, bit reactive and packet reactive jammers by analyzing bit errors in each packet and signal strength. Once a node detects a jammer it sends an alarm messages to their neighbors. This perspective of sending an alarm message is interesting because in our proposal, when a node is attacked because its performance metrics drastically changed values, it sends a control packet. This way the network can isolate the jammer node.

In [17], the jammer detector examines packet delivery ratio (PDR) [7] and received signal strength indicator (RSSI). In [18] the authors inspect Signal Strength, Autocorrelation of Signal Strength, Packet Delivery Ratio to detect jamming. In [19], the authors use throughput and packet drop ratio to detect a jammer attack. In these works, jammer presence analysis is performed based on known and related metrics to signal strength. Our work complements this perspective by analyzing routing metrics to give more power to the decision to isolate a node from the network.

Burbank et al. in [20] studied path statistics and spectrum availability for the jammer detector. In [21], the authors assume a jammer that constantly emits signals, which can also attack several channels at the same time. The jammer detector is based on load balancing. This load balancing perspective is quite interesting and could be a future addition to our proposal when analyzing different traffic rates and the network is more versatile and dynamic.

### 3.2. Mitigation through Routing

In [22], the quality of the channel is evaluated using: signal-to-noise ratio, a cost of links, data dropped rate and time. Once the jammer is detected, then AODV protocol is adapted to avoid routes in the attacked area. The scope of their work was limited to AODV. Our proposal complements the perspective of modifying the AODV protocol and we added another widely used protocol such as DSR. Here the authors modify the routing of the protocol, in our proposal we modified the protocol adding a control packet based on values of performance metrics.

In [23] Sheikholeslami et al. measure constant jamming level, and the routing algorithm uses this information to decide the best routes. Since they are limited to constant jamming they only analyze energy to detect it. Our work further deepens the performance parameters to detect an area under jamming, such as: delay, retransmissions, traffic, overhead, among others. In [24] Mustafa et al. authors detect one or more stationary and mobile jammers and with this information define multi-path to transmit packets with a modified AODV, which analyzes the reliability in routes to decide the next hop. Their work was also restricted to AODV.

In [25], the metrics used to detect jamming are: signal-to-noise ratio, transmission power, and delay. The routing algorithm employs information of jamming detectors to select the best route. In their work, they do not isolate the area under attack, and when the jamming area changes the selected route could be inside of this new jamming area degrading the performance of the network. Authors only use throughput to evaluate the performance of the network. It would have interesting if the authors have also measured the performance of the network using the energy consumption in the nodes, and the resilient when jamming conditions change. In the work cited in [26], they propose a comparison under security conditions between protocols on-demand and proactive protocols. The authors introduce an attack and test reactive routing protocols widely known in the literature as AODV and DSR. They analyze a Secure Neighbor Detection scheme and integrate it with an On-Demand Protocols: AODV and DSR. They implement 100 nodes in a 1000 × 1000 array under a defined topology and study metrics such as valid routes and overhead. As in our work, the authors have a fixed topology in order to control the system variables, however, we propose a greater number of performance metrics in order to have a broader perspective of the effects on the network before the attack and after the mitigation scheme. Due to the fact that ad hoc mobile networks constantly change, this makes them vulnerable to attacks, specifically jamming. In [27] the authors analyze performance for known protocols in sensor networks and study the impact of jamming and propose a way to mitigate it. One of the main problems in networks with mobility and unstable links is the routing of information. That is why, as in our work, improving the efficiency of the routing protocol is a great challenge for communications. The authors analyze Physical and Virtual Jamming and perform a bibliographic review of the attacks and routing protocols studied in wireless networks, and here they include AODV and DSR protocols.

## 4. Proposed Mitigation Mechanism for Jamming Attacks

We propose a two-phase mechanism for mitigation. The first phase concerns the detection of those nodes with communications that are being directly exposed to jamming. The second phase consists of a reconfiguration of the routes to avoid the affected nodes.

The rationale behind the proposed scheme is that if we only exclude the jammed nodes, then the overall performance of the network may be reduced. However, the performance can be improved by reconfiguring the routes with the unaffected nodes. In this way, we avoid the propagation of the effects of the jamming and the waste of bandwidth and energy due to the packet re-transmissions and overhead of the routing protocol.

Since a WSN has very limited energy resources, and jamming may cause waste of energy, therefore, we will also analyze the energy consumption of the nodes and it is expected that the proposed mitigation scheme reduces the waste of the energy associated due to the jamming.

The detection algorithm is designed for applications where the sensor networks is periodically time-sampling different part of a large process, where each sensor can be configured to sample in the range of 1 to 10 s. At the beginning, the sensor network is calibrated for a specific process and once calibrated it will remain like that; therefore, the traffic conditions generated for the sampled information is very stable. However, since the environment where the sensor network is going to work is diverse, we train the metrics of the detection algorithm to adapt to this environment (attack-free scenario). For example, if the network is in a harsh environment, then this will be the normal operating conditions, and the metrics would be markedly different to the metrics in a cleaner environment. Then, the training step reflects the normal conditions and it will be used to detect anomalies in the network. In addition, the proposed detection scheme in its testing phase has an epsilon constant, which could be adjusted to reduce errors and can be selected depending on the environment and error in the performance metrics. Besides, by using three parameters of evaluation we reduce false positives which can reinforce the good method operation under non-ideal conditions.

### 4.1. Mitigation of Jamming Attacks Criteria

Detection of nodes under jamming: Each node is running a local-based decision mechanism to detect if jamming is affecting its communication parameters. Control packets are sent to notify its neighbors when jamming is detected in order to eliminate it from their routing table as a route.

Routes reconfiguration: Once a node under jamming attack has been identified, it will be removed from the neighbor tables of the nodes that received the control packet. The self-configuring mechanism of the routing protocol will adjust the routes for excluding the jammed nodes.

### 4.2. Detection of Nodes under Jamming

A node that is experiencing a jamming attack might detect such condition by using a heuristic anomaly model. In this way, the node observes a set of variables which are compared to the values of the same variables under normal conditions (without attack).

The jamming detection model considers energy consumption, an average number of packet re-transmissions and the average number of retrials in the inner loop of the CSMA/CA algorithm. These three metrics have previously shown to be affected by jamming conditions [10,11].

First, we create a network’s metrics model by training each metric with a database of the observed values obtained when the network was working under an attack-free scenario. Our model is trained locally for each node as follows; we compute the range $\left[v_{min},\;v_{max}\right]$ for a given variable *v*, where the *k*-th training value of *v* is denoted as $v_{k}\in \left[v_{min},\;v_{max}\right]$, see Algorithm 1. For reducing the number of false positives we have smoothed the input data with a moving average filter of length *m*, where the output of the filter is the training value $v_{k}$ of the detection algorithm, this filtering is also done with the testing data.

**Algorithm 1:** Training of the detection algorithm.**for** each node **do** (a) moving average filtering of input data; (b) compute the min and max value of each variable: Energy, Retransmissions, CSMA-retries.**end for**


For testing the detection model, a decision algorithm is run locally in each node, the *i*-th test value $v_{i}$ of *v* is compared to its corresponding $v_{max}$ value, if $v_{i}>v_{max}+\epsilon$, where ϵ is a constant, then the node could be under a jamming attack. In order to reduce the false alarms we use a majority vote rule, i.e., an indication of jamming is produced when a majority (two or three) of the variables considered in the model exceeds the corresponding thresholds. Once the jamming detection algorithm produces a positive result, then a notification of jamming attack is emitted to the neighbor nodes by sending a control packet, see Algorithm 2.

**Algorithm 2:** Testing of the detection algorithm.**for each** node **do** n=0; ϵ:constant>0; moving average filtering of input data with 10 s of step; **for each** variable: Energy, Retransmissions, CSMA-retries. **do**  **if** test value exceeds max +ϵ **then**   n++;  **else**   DO nothing;  **end if** **end for** **if** n≥2 **then**  jamming alarm:  SEND control packet for notification; **else**  DO nothing; **end if****end for**


### 4.3. Routes Reconfiguration

To isolate the nodes affected by the jamming from the network, the routes reconfiguration should be done. This reconfiguration step depends on the routing protocol, either ADOV, DSR or MPH. Next, we will explain how these protocols work.

AODV is a reactive protocol that sends hop by hop packets. The routes have an expiration time and this protocol represents a decrease in the processing of nodes, decreases the expense in memory and reduces control traffic. In order to keep only the most efficient routing information, AODV has the concept of sequence number for packets. This number is an integer value that each node increments before generating a control message to copy it to the packet before sending it. The lifetime of the route is the time the route expires or must be deleted. This prevents lost packets to be traveling on the network and using links whose status has not been known for a long time. AODV has the advantage of repairing small faults in the network before notifying to the others nodes, in order to avoid using bandwidth [28].

DSR is based on source routing. Nodes maintain caches, whose entries include the destination and the list of nodes to reach it. This protocol consists of two main mechanisms: path discovery and route maintenance. The source node is responsible for discovering the route from each source node to the destination node. This can generate a bit of latency if the packets have too long headers for routes. Once the route is established, then maintenance mechanism starts. The latter steps consist in to keep monitoring the error of the received packets in each node of the route [28].

MPH is a proactive, hierarchical multi-parent protocol that establishes links based on node hierarchy, starting from the highest hierarchy node, which is the coordinator node. This type of protocol has fewer links per node, then the other routing protocols because it only allows parent-child links, but does not allow links between nodes with the same hierarchy level. Path reconfiguration is performed by HELLO packets that are sent periodically in order to check if the neighbors of a node still exist. If no response is received from these nodes, they are removed from the neighbor table and are no longer used as valid routes. This is where network topology changes because hierarchy levels of the nodes change.

When a notification of jamming is received by node *i* from node *j*, node *i* will adjust its neighbor table removing node *j*, thus *j* is not considered as a neighbor of *i* and a reconfiguration of routes in the network will be carried out by the routing protocol.

**Figure 2:**
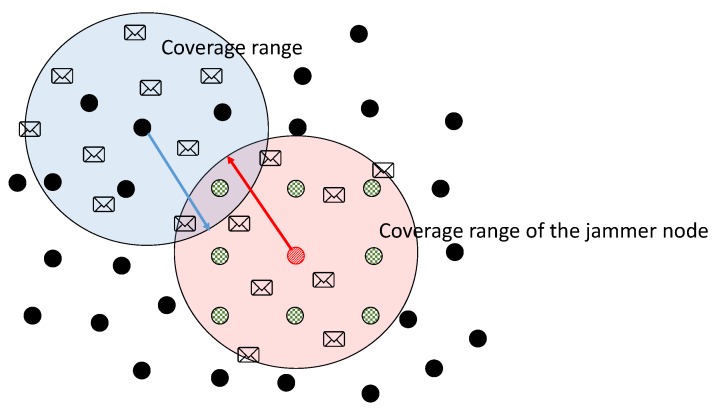
Coverage range of the jammer node and its influence in its neighbors.

Figure 2 shows the coverage radii of two nodes in the network. We take as an example the radius of the jammer node and the radius of an arbitrary node in the network. We can observe that when there is a positive jamming detection, the node sends a control packet that causes its neighbors to remove it from their neighbor tables. In this way, according to the coverage radius, as a hypothesis, we can argue that nodes surrounding the jammer node will be removed from the network, causing reconfiguration of routes. This is because these nodes, according to the graph, can lose packets coming from other nodes that are not directly affected by the jammer node. The effect of removing a node as a neighbor is that it reduces the probability of having a high packet loss.

Reconfiguration of routes in the WSN is carried out differently for each of the protocols considered in this paper. When routes change, due to the presence of a malicious node, AODV runs a mechanism of error messages and also puts in each created route an expiration time (Time To Live). If there is a node that does not respond during that time, the route is taken as invalid and becomes obsolete. The DSR protocol only handles error packets for route maintenance. The DSR routing protocol was specifically designed to be used in Mobile Ad hoc Networks (MANETs) and makes the network self-organizing and self-configurable, and implements two mechanisms to discover and maintain routes from source to destination: Route Discovery and Route maintenance. The AODV routing protocol was designed for Ad Hoc networks and only maintains the route information that needs a node to determine the next-hop, but not the entire route; it is based on the information that nodes have about their active neighbors. The mechanisms in AODV used to discover and maintain the routes are: Route request, Route reply, Route error and Route reply. In contrast, the MPH protocol is a proactive protocol that does the reconfiguration of routes through hierarchies, starting from a root node. In this way, nodes establish new links by exchanging hierarchy values to establish relationships between parents and children. The maintenance of routes is carried out by discovery packets of neighbors for each period of time previously established.

Modifications for the AODV and DSR protocols resulting in the modified AODV-M and DSR-M protocols are shown in Algorithm 3. Where AODV is when the Route Discovery is Hop-by-Hop and DSR is Source Routing; AODV-M and DSR-M is reflected in the Detection testing algorithm step.

Modifications for the MPH protocol resulting in the modified MPH-M protocol are described in Algorithm 4. Again, MPH-M is reflected in the Detection testing algorithm step. 

**Algorithm 3:** The proposed modified protocols: AODV-M and DSR-M.**for each** node *i* **do** Node *i* turns on; SEND HELLO packet; RECEIVE ACK packet; Detection testing algorithm (Algorithm 2); **if** TRAFFIC packet **then**  **if** Node *i* does not have this route **then**   Route discovery   SEND RREQ packet;   RECEIVE RREP packet;  **else**   Route maintenance   SEND TRAFFIC packet;  **end if** **end if** **if** a CONTROL packet is received from Node *j* **then**  REMOVE Node *j* from neighbor table; **else**  DO nothing; **end if** **if** a route expires **then**  SEND RERR packet; **else**  DO nothing; **end if****end for**


**Algorithm 4:** The proposed modified protocol: MPH-M.**for each** node *i* **do** Node *i* turns on; Discovery.time=0; SEND HELLO packet; RECEIVE ACK packet; Detection testing algorithm (Algorithm 2); **if** Discovery.time%10==0 **then**  **if** Node *i* does not have this route **then**   Route discovery;   SEND HELLO packet;   RECEIVE ACK packet;  **else**   DO nothing;  **end if** **end if** **if** TRAFFIC packet **then**  **if** Node *i* does not have this route **then**   SEND TRAFFIC packet to its parents;  **else**   SEND TRAFFIC packet to specific parent;  **end if** **end if** **if** a CONTROL packet is received from Node *j* **then**  REMOVE Node *j* from neighbor table; **else**  DO nothing; **end if** Discovery.time++;**end for**


## 5. Anomaly-Based Model

The variables used for the Normal Behavior model are energy, number of re-transmissions and number of retries of the inner loop of the MAC algorithm. In this section, therefore we are interested in their behaviors in both, normal and jamming conditions and we analyze how discriminant they are. For simulations, the length of traffic packets is 100 bytes. Regarding the propagation model, sensitivity thresholds are between −78 to −94 dBm, and transmission power is 4.5 dBm in active mode.

### 5.1. Scenarios of the Experiments

**Figure 3:**
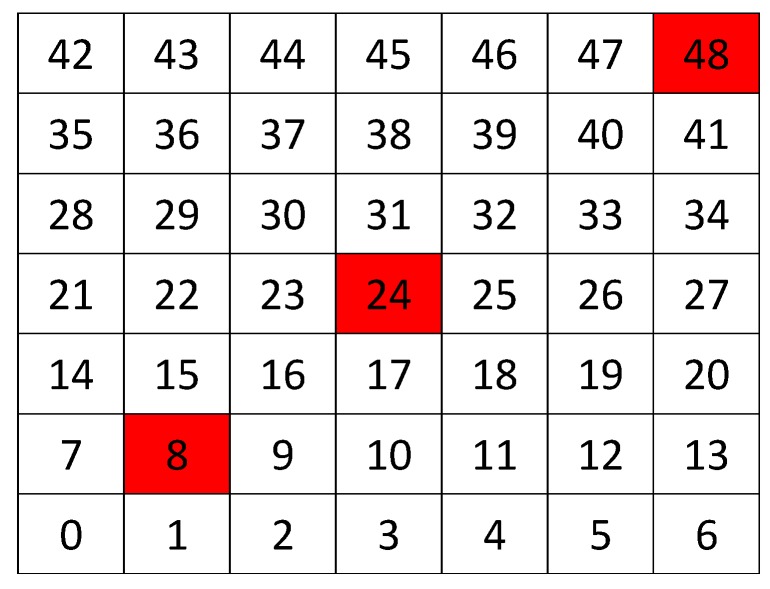
Network grid.

We have simulated a network with 49 nodes, physically arranged as a grid topology of 7 × 7 nodes with 5 m between each node and its neighbor horizontally and vertically, see Figure 3. This is because we would have a specific topology and we can play with positions of the jammer node and co All nodes in the network has the same traffic rate (10 packets per second in each node). Packet loss in the links is between 0.5–1.5%. We have used the standard IEEE 802.15.4 for the MAC layer. This standard describes very low energy to work. On the other side, data transmission rate is very low. The energy model presented in this analysis is the model studied in [11]. It consists of starting, microcontroller energy, transmission energy, receiving energy, switching energy, algorithm CSMA/CA energy, and shutting down energy. These are the main tasks that a node executes on the network and are considered in active mode, without sleep times.

Table 1 describes the parameters of the simulations. We use an event-driven simulator based on C++ language designed and implemented by us, proven in [29]. This is a network simulator with parameters of MAC and Network layer, and it has punctual features of the Physical layer such us: percentage of interference in the channel and RSSI (Received Signal Strength Indicator) parameters.

### 5.2. Distinguishing Normal from Jamming Conditions

Once we have selected the set of performance variables for the anomaly model, it is important to perform an analysis of such variables to determine if they can be useful for the detection of the jamming attack. Ideally, only those nodes affected by the jamming should present significant deviations in the variables used in the model; thus, other nodes that are not neighbors of the nodes under jamming would not have relevant changes in the behavior of the observed variables; however, in practice not all the variables may exhibit such tendency, therefore a majority voting rule for the detection algorithm will be later considered.

**Figure 4:**
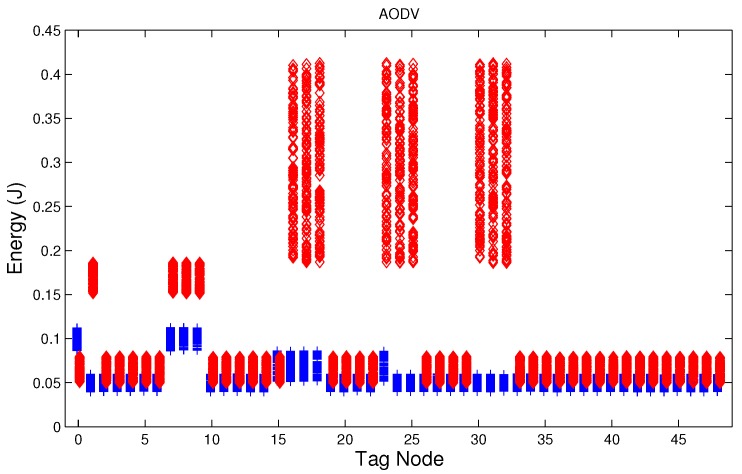
Energy consumption of the nodes from Figure 3 with AODV (Ad hoc On Demand Distance Vector) under normal (blue) and jamming (red) conditions.

**Figure 5:**
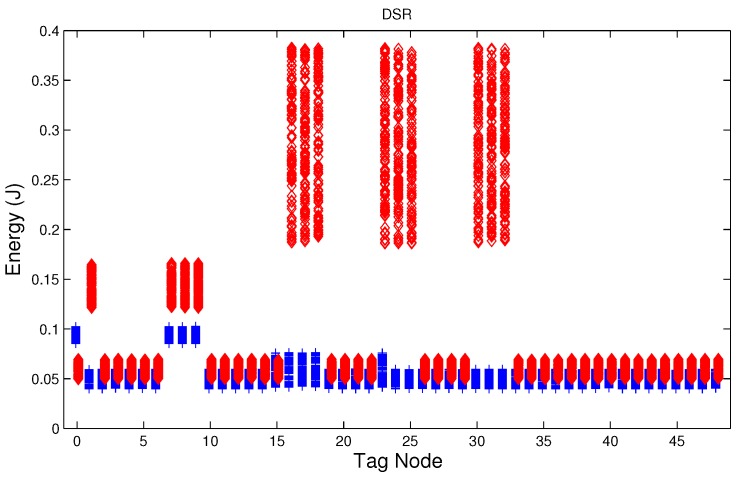
Energy consumption of the nodes from Figure 3 with DSR (Dynamic Source Routing) under normal (blue) and jamming (red) conditions.

**Figure 6:**
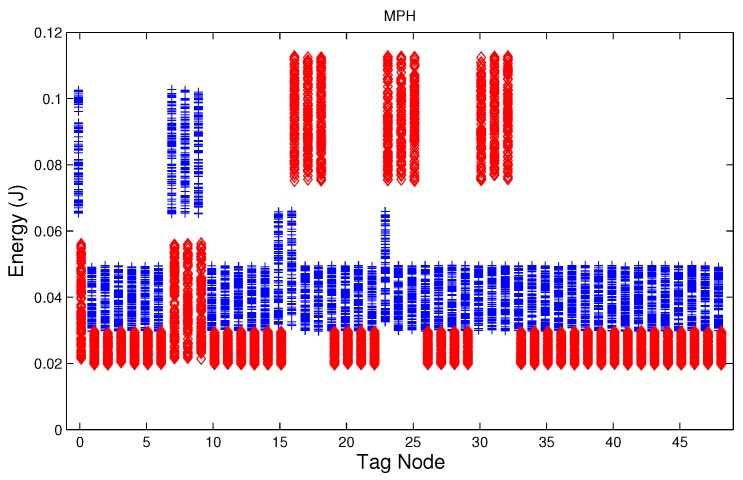
Energy consumption of the nodes from Figure 3 with MPH (Multi-Parent Hierarchical) under normal (blue) and jamming (red) conditions.

**Figure 7:**
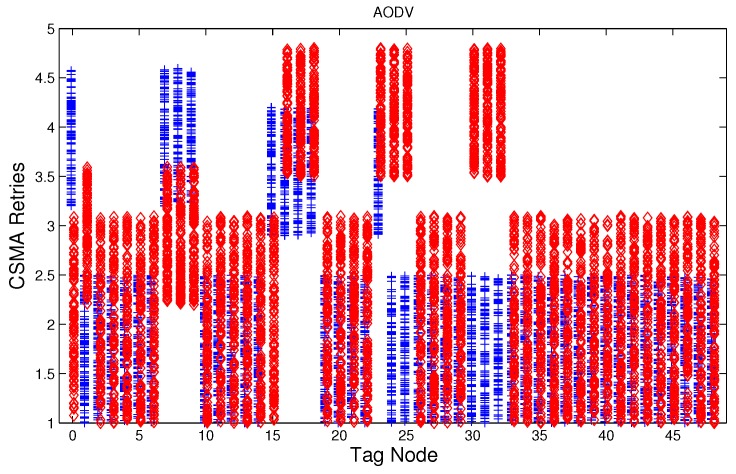
Average number of retries (MAC algorithm) for the nodes from Figure 3 for AODV (Ad hoc On Demand Distance Vector) under normal (blue) and jamming (red) conditions.

**Figure 8:**
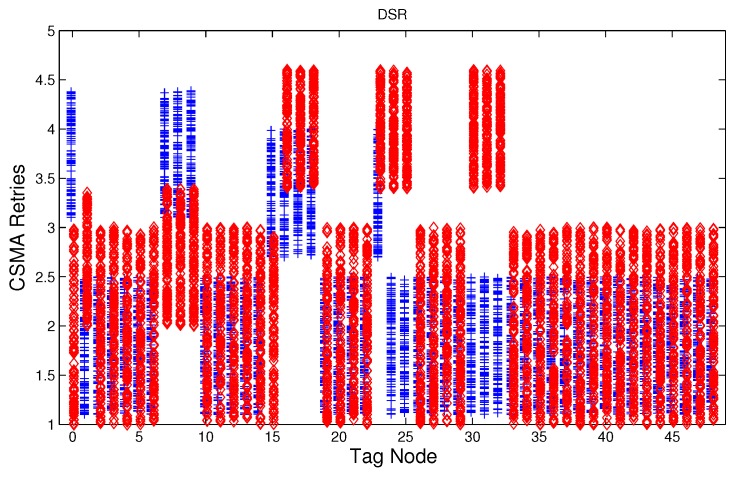
Average number of retries (MAC algorithm) for the nodes from Figure 3 for DSR (Dynamic Source Routing) under normal (blue) and jamming (red) conditions.

**Figure 9:**
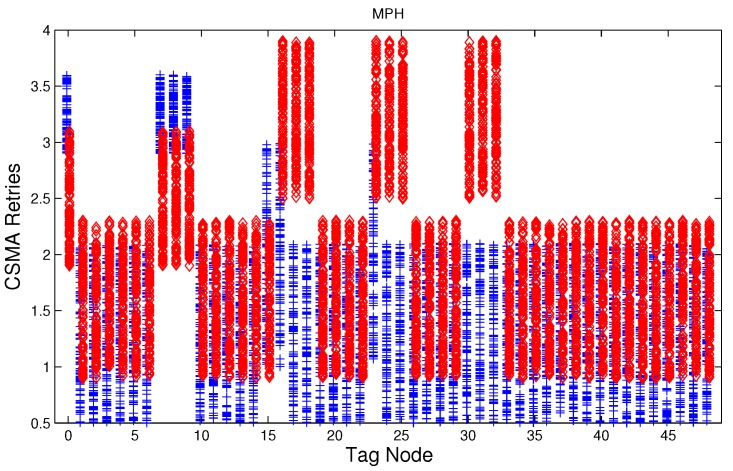
Average number of retries (MAC algorithm) for the nodes from Figure 3 for MPH (Multi-Parent Hierarchical) under normal (blue) and jamming (red) conditions.

**Figure 10:**
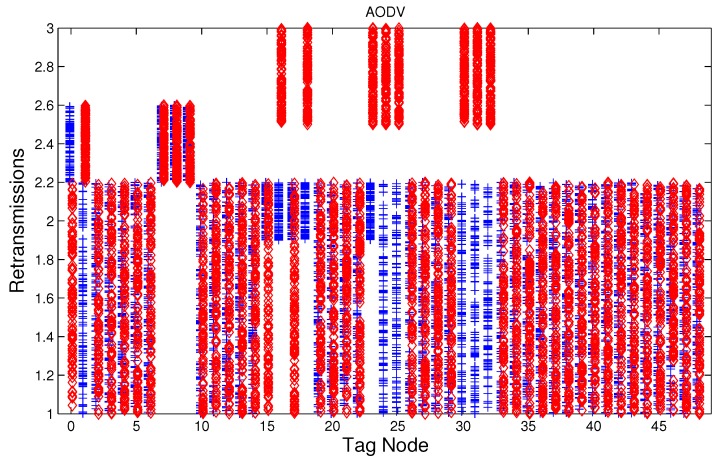
Average number of packet re-transmissions of the nodes from Figure 3 for AODV (Ad hoc On Demand Distance Vector) under normal (blue) and jamming (red) conditions.

**Figure 11:**
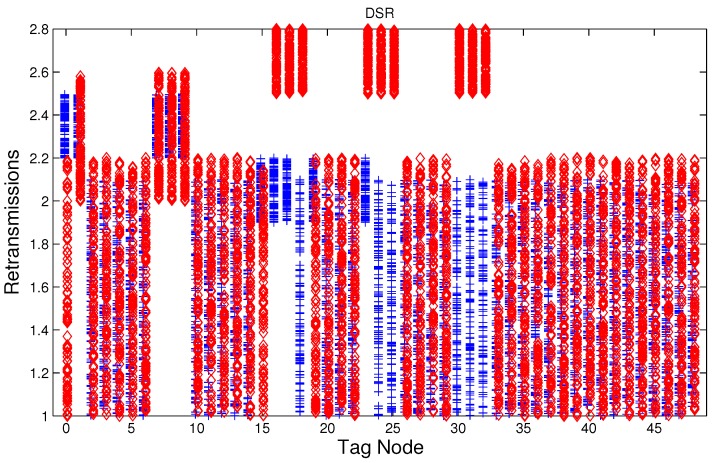
Average number of packet re-transmissions of the nodes from Figure 3 for DSR (Dynamic Source Routing) under normal (blue) and jamming (red) conditions.

**Figure 12:**
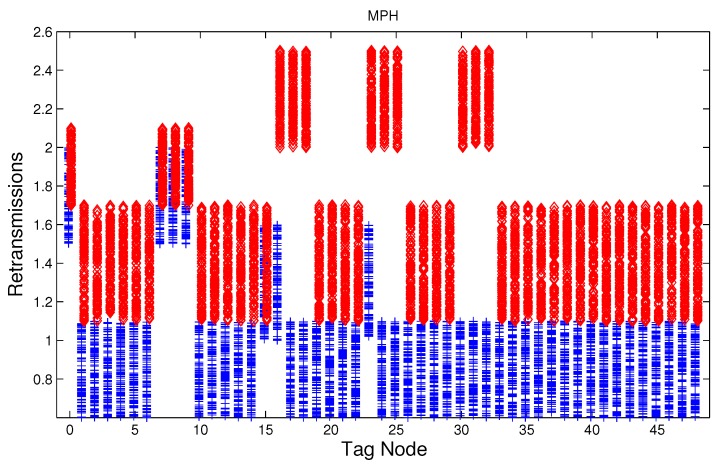
Average number of packet re-transmissions of the nodes from Figure 3 for MPH (Multi-Parent Hierarchical) under normal (blue) and jamming (red) conditions.

We have studied attack-free and jamming scenarios with a jammer located at the center of the grid (above node 24). The simulated time is 100 s, each variable is sampled every second and the values are displayed in Figure 4, Figure 5, Figure 6, Figure 7, Figure 8, Figure 9, Figure 10, Figure 11 and Figure 12, which show the results of the performance metrics for each node of the network. Blue points represent the values for normal condition and red ones are for the jamming conditions.

For instance, Figure 4 shows the sampled values of the energy consumption for each node. Each node is tagged with an integer ranging from 0 to 49, this tagging corresponds to the network grid presented in Figure 3. We have obtained in total 200 sampled values in the simulations for each node, 100 values were obtained simulating normal conditions and 100 under the jamming attack. Such 200 resulting samples are drawn according to their values in Figure 4 in a vertical alignment above the tag for each node, Figure 5, Figure 6, Figure 7, Figure 8, Figure 9, Figure 10, Figure 11 and Figure 12 were built following the same construction method. From this graphical representation can be seen where a significant deviation of the performance variables occurs, which can be related to the jamming attack. A larger separation of the blue points from the red points result in a better distinction of an attack from a normal behavior. Since the attack here considered was centered in the node with tag 24, then ideally those nodes nearby with tags 16, 17, 18, 23, 25, 30, 31 and 32 are also expected to be affected by the behavior of the variables of the model. In the following subsections, we will study how each variable behaves using Figure 4, Figure 5, Figure 6, Figure 7, Figure 8, Figure 9, Figure 10, Figure 11 and Figure 12.

### 5.3. Energy in the Node

During a jamming attack, the energy wasted by each node increases. Figure 4, Figure 5 and Figure 6 show that for all routing protocols, AODV, DSR and MPH, the energy consumption is a suitable metric for deciding if the node is under attack. We can observe that for AODV and DSR most of the nodes of the network increase their energy consumption under the presence of a jammer; particularly those nodes located in the jamming area (nodes labeled as 16, 17, 18, 23, 24, 25, 30, 31, and 32) show a higher level of energy consumption which can be used as indication of anomaly behavior. Moreover, these figures show that for MPH the number of nodes affected due to jamming is lower than the ones affected in AODV and DSR.

### 5.4. CSMA/CA Retries

Before a node starts transmitting a packet, the MAC layer listens the channel, if the channel is busy since there is an ongoing transmission, then it waits for a random time, otherwise, it transmits the packet. In order to reduce the probability of collision of other nodes that have been waiting for an idle channel, a backoff algorithm generates another random waiting time, with a uniform distribution probability defined over an interval named as contention window. After this time finishes, then the node sense the channel again, and this loop can be repeated if the channel is busy. The number of times the loop is done is referred in this work as retries. The standard IEEE 802.15.4 [2] limits the number of retries to five, and therefore we use such maximum number of retries.

Figure 7, Figure 8 and Figure 9 show that for AODV, DSR and MPH, the implementation of the average of the number of retries is also a good metric to decide if the node is under attack. It is observed that most of the nodes of the network increase their number of retries under the presence of a jammer for AODV and DSR; particularly those nodes located in the jamming area show a higher number of retries, which can be related to an anomaly behavior.

### 5.5. Retransmissions

The MAC layer starts a timer with a value $t_{w}$ seconds when node *i* sends a data packet to node *j*, if an acknowledge (ACK) packet from node *j* arrives before the timer expiration then a successful delivering of the data has been carried out and no further action of the node *i* is needed, if the ACK packet does not arrive during the waiting time then data packet is retransmitted, and the MAC layer starts again a timer with a value $t_{w}$. According to the IEEE 802.15.4 standard, we set three as the maximum number of retransmissions. The average number of retransmissions for AODV, DSR, and MPH is depicted in Figure 10, Figure 11 and Figure 12, from that it can be concluded that the use of the average of the number of retransmissions may be another suitable metric for jamming detection. We can observe that for AODV and DSR, most of the nodes of the network increases the number of retransmissions under the presence of a jammer; in a similar way to the other metrics mentioned above, retransmission are taken into account for the anomaly model.

### 5.6. Performance of the Anomaly Model

Table 2 shows the results of testing the detection model, those nodes that are under jamming must detect such condition and send a notification packet. In these simulations the jammer node is located in the middle of the topology (above node 24), therefore nodes labeled as 16, 17, 18, 23, 24, 25, 30, 31, and 32 must detect the jamming attack when the detector is ideal. The simulation time for training and testing is 100 s, and we observe the nodes that have a positive detection of jamming every 10 s in the testing phase. This means that in these nodes the number of variables with values higher than the thresholds permitted by Algorithm 2 were two or three (n≥2) and this may be an indication of an attack condition. For these simulations, the input data is smoothed by a moving average filter of length 10, see Algorithms 1 and 2. For simplicity we have used ϵ=0, a higher value of ϵ may reduce the number of false positives but it would degrade the detection rate. The results obtained are 100% of true positives for all the routing protocols and 8.25%, 9.0% and 1.75% of false alarms for AODV, DSR, and MPH, respectively.

## 6. Results

**Figure 13:**
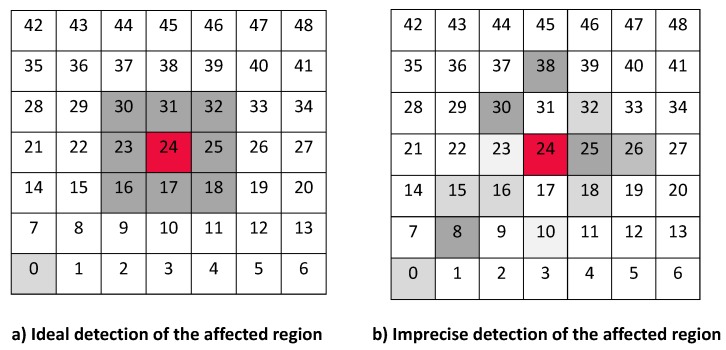
Difference between ideal detection and imprecise detection of a jammer node.

For this experiments, we expose the scenario described in Section 5.1, and we set the collector node is in the lower left corner and we have three different positions for the jammer node, depending on the case: near (When the jammer node is in position 8 in Figure 13), middle (when the jammer node is in position 24), and far (when the jammer node is in position 48). Throughput is calculated as a function of the traffic rates. We develop some experiments using an ideal channel and noisy channels. We also test the performance of the algorithms under jamming. The simulation is repeated 10 times, and the tables show the average of these simulations.

In our experiments, we have defined that we only will have a jamming attack at the same time. We also define three positions where this attack can occur; Figure 3 shows a grid, where each square represents a node, then the three possible position where a jammer attack can occur are in nodes for positions 8, 24 and 48. When the attack occurs at node 8 we call it the “near attack”, because the attack is near the collector node. When the attack is at node 24 we call it the “middle attack”, because the attack is middle of the network. Finally, when the attack occurs at node 48 we call it the “far attack”, because the attack is very far of the collector node.

### 6.1. Jamming Attack Detection Scheme

Table 3 shows the areas bounded by the nodes in each position of the jammer. For each protocol, the table displays which nodes are free of attack (with a ✓) and the nodes that are under attack (with a ×). From this table, we can observe that the MPH-M protocol makes a better delimitation of the affected area, around 95% of success, because almost all nodes that should form the area were included in it.

The Table 3 shows the result of the jamming detector for those nodes that are under attack. The table exposes different results of detection for each of the studied protocols, this is because each routing algorithm has different design principles and therefore the performance of the detection mechanism will also differ for each case. We note that MPH-M has a better performance against detection. For the case when the jammer node is near the collector node, MPH-M has an efficiency in detecting the affected nodes of 88.9%, AODV-M has 44.4% and DSR has 88.9%. For the case when the jammer node is in the middle of the topology, MPH has an efficiency of 100%, AODV-M has 77.8% and DSR-M has 88.9%. For the case where the jammer node is far from the collector node (the opposite corner in the topology), MPH has an efficiency of 100%, AODV has 75% and DSR has 100%.

### 6.2. System Throughput

**Figure 14:**
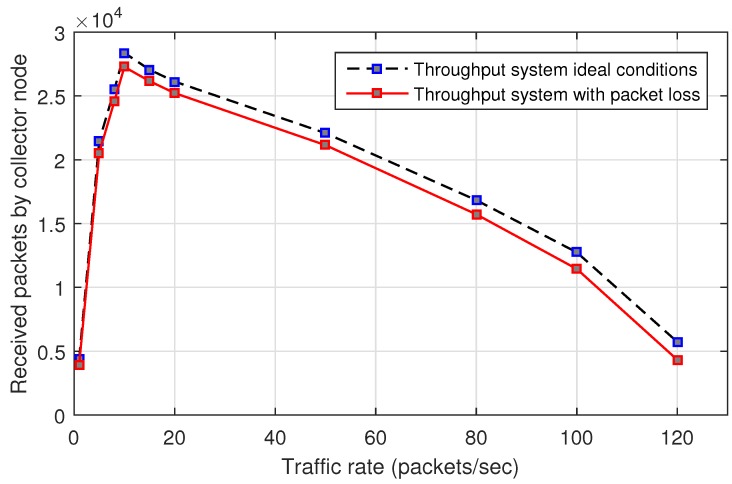
System throughput.

Figure 14 shows the system throughput for a network with jamming-free conditions, traffic packets are sent by the nodes to the collector node under MPH protocol. There are two scenarios: In one of them, we have ideal links with zero packet loss, i.e., packet loss can only be caused by collisions. The other one considers the value of packet loss in the links different to zero. The simulation time is 100 s. There are no considered copies packets (same packets of the original packet). We may observe that the maximum throughput is achieved when all nodes transmit at 10 packets/s; for transmission rates higher than this value the throughput is smaller because the collisions increase; for transmission rates lower than this value the throughput is lower because the amount of data is small. We can work in the interval before the peak.

Table 4 shows the percentage of retransmission packets for the MPH protocol for each of the studied traffic rates. This table shows how the percentage of packets to be copied due to retransmissions increases, as the traffic rate of the nodes and collisions increase. Here simulations are performed in ideal conditions with respect to the links. However, packet collisions can occur. This is important because here we can observe the number of packets to be re-transmitted because no acknowledgment was received at the destination node. This metric complements Figure 14 under ideal conditions in order to clarify that ideal throughput also considers packet collisions.

**Figure 15:**
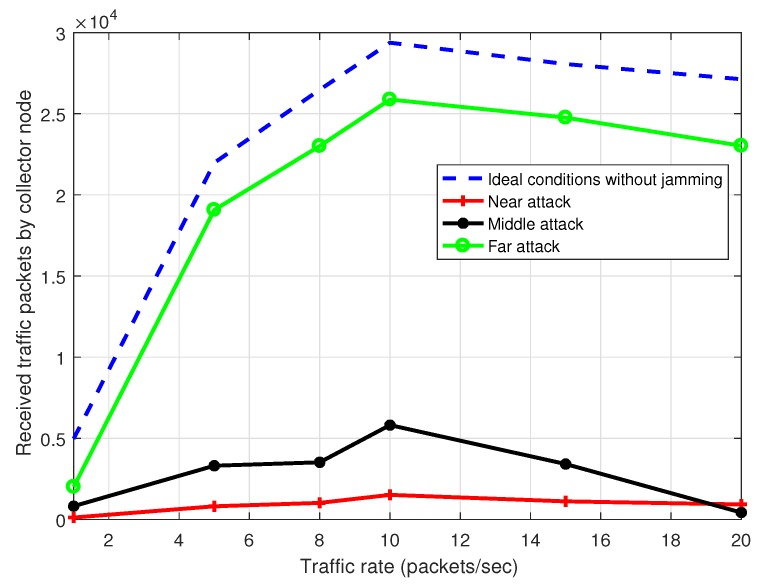
System throughput with ideal conditions and under attack with the jammer node is located near, in the middle and far from the collector node.

In Figure 15 we observe the system throughput curve in presence of jamming with ideal packet loss in the links under MPH-M protocol. The simulation conditions are the same as in Figure 14. However, this latter figure shows the throughput when the jammer node is near to the collector node, when it is in the middle of the topology and when it is far from the collector node. Besides, we observe the throughput with ideal conditions without jamming. We note that when the jammer node is close to the collector node, there is almost no packet reception due that a bottleneck is formed near the collector node. Also, the figure shows that a position that causes a lot of damage is when the jammer node is in the middle of the topology, because the number of affected nodes is high.

**Figure 16:**
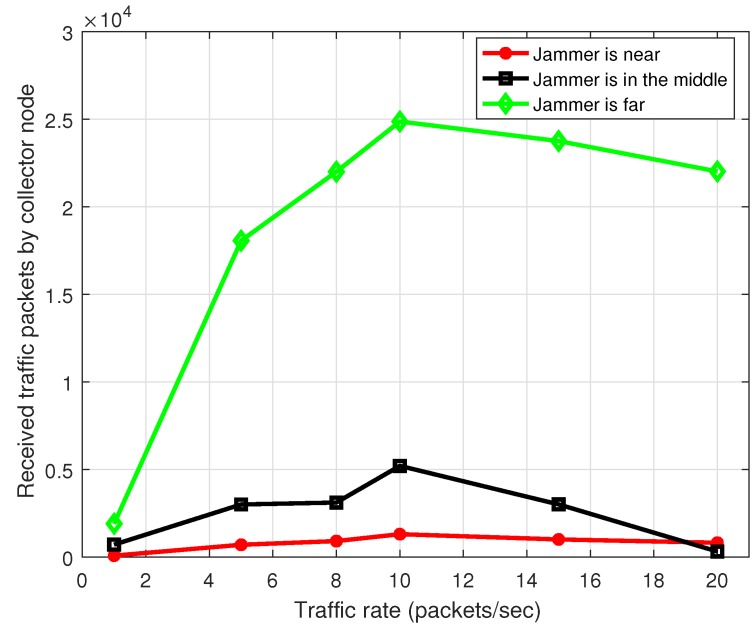
System throughput with jamming, the jammer node is located near, in the middle and far from the collector node. Links have packet loss.

Figure 16 presents the throughput for MPH under non-ideal conditions . Links have packet loss due to channel interference and communication problems. Here we see how is the change of the number of received packets by the collector node. When the conditions of links among nodes are ideal, as in Figure 15, there is a difference of about 10%, where the throughput under non-ideal conditions is getting worse and about 10% more packets that are lost than under ideal conditions.

**Figure 17:**
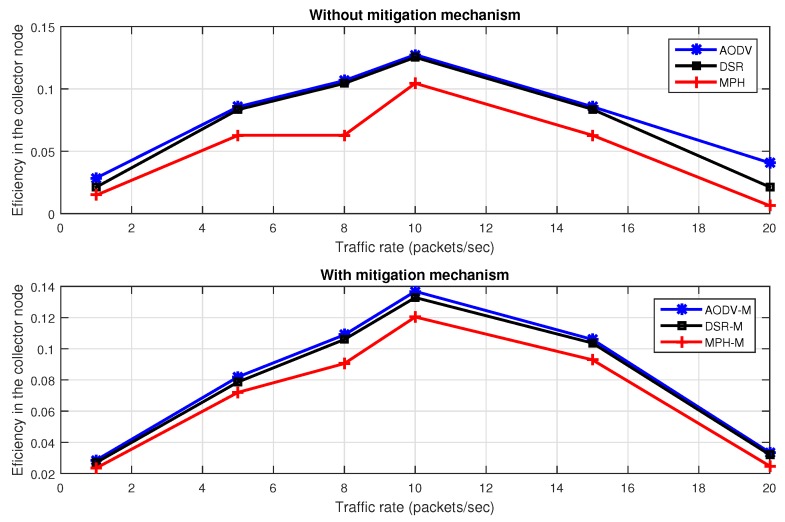
Throughput for different data rate for AODV, DSR, MPH, AODV-M, DSR-M and MPH-M under jamming.

Figure 17 describes efficiency with different packet traffic rates with the jammer node in the middle of the topology, with and without the proposed mitigation mechanism. We note that the MPH-M protocol is 17% more efficient without the mitigation scheme, and 8% more efficient with the mitigation scheme to support traffic packets with respect to AODV-M and DSR-M protocols.

**Figure 18:**
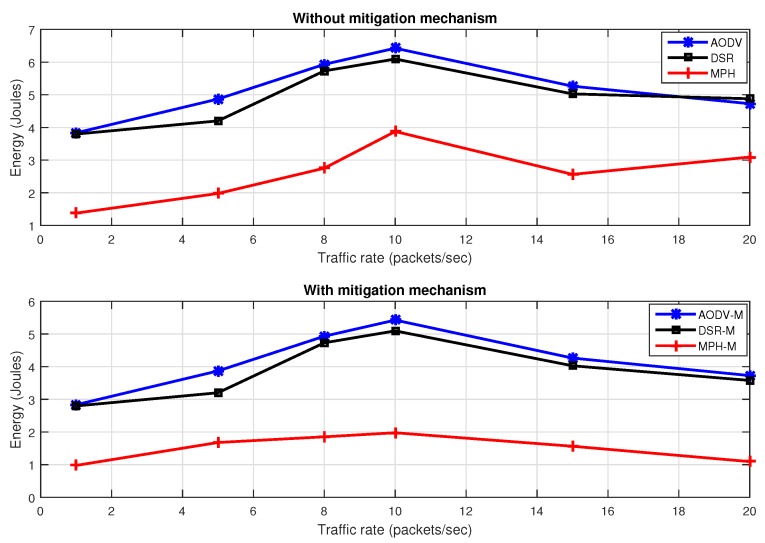
Energy consumption for different data rate for AODV, DSR, MPH, AODV-M, DSR-M and MPH-M under jamming.

Figure 18 shows energy with different packet traffic rates with the jammer node in the middle of the topology, with and without the proposed mitigation mechanism. We note that the MPH protocol has 39% less energy consumption without the mitigation scheme, and MPH-M uses 64% less energy with the mitigation scheme with respect to AODV-M and DSR-M.

Table 5 describes values of the main performance metrics per node under an uniform random traffic rate generation. Traffic rate on the nodes is between 1 and 10 packets per second. The topology has non-uniform random distribution. Under this scenario, regarding the retransmissions, MPH-M improves in 11% than MPH, and under mitigation scheme is better in 12% against AODV-M and DSR-M. With regard to the CSMA retries, MPH-M exhibits an average reduction of 6% against MPH and against AODV-M and DSR-M of 11%. Concerning overhead, MPH-M has 29% less than MPH and 44% compared to AODV-M and DSR-M; this metric shows the proactive nature in the MPH protocol. Regarding delay end-to-end, MPH-M is faster 18% than MPH and 50% than AODV-M and DSR-M. Finally, on energy, MPH-M has energy savings of 4% compared with MPH and 37% against AODV-M and DSR-M.

The Table 6 shows values of performance metrics in the network. The tests were made with non-ideal links and traffic rate of 10 packets per second per node under the grid topology. Metrics are average values of re-transmissions and CSMA retries. The Delay end-to-end is calculated as the average per route. Overhead is a metric that influences the amount of network collisions and the channel occupancy. It depends on the number of routing packets that the routing protocol needs to connect to nodes and to route packet traffic. Thus, it is calculated taking into account the number of control packets that are needed to route a traffic packet. Ideally, a routing protocol should need the least amount of control packets. Metric such as Energy is given by the energy per node in the network.

We can observe that MPH and MPH-M are more efficient, for all metrics, than AODV and AODV-M, and DSR and DSR-M and MPH. For example, in Figure 18 we can observe that the total energy for MPH is 192.57 J and 312.13 J and 294.98 J for AODV and DSR respectively. Also, with respect to the energy per node, MPH has around 3.93 J, AODV has 6.37 J, and DSR has 6.02 J. Regarding the mitigation mechanism, in Figure 18 we can observe that the total energy for MPH is 102.8 J and 220.32 J and 214.97 J for AODV-M and DSR-M respectively. Also, with respect to the energy per node, MPH has around 2.57 J, AODV-M has 6.12 J, and DSR-M has 5.81 J. Table 7 shows how much more efficient the performance metrics are for MPH-M than other protocols, when the network is attacked. This table helps to visualize improvements among the protocols. We can observe that for most of the metrics the MPH-M better adapts to the mitigation scheme, than AODV-M and DSR-M.

Moreover, we can also note that under attack, the metrics improve when the mitigation algorithm is used. Table 8 shows how each protocol improves each metric when mitigation scheme is used. We can observe that for all metrics, the MPH adapts much better to the mitigation scheme. For example, with respect to the CSMA retries, MPH-M improves 32.27% compare with only 32.27% and 39.04% for AODV-M and DSR-M, respectively.

### 6.3. Resilience

**Figure 19:**
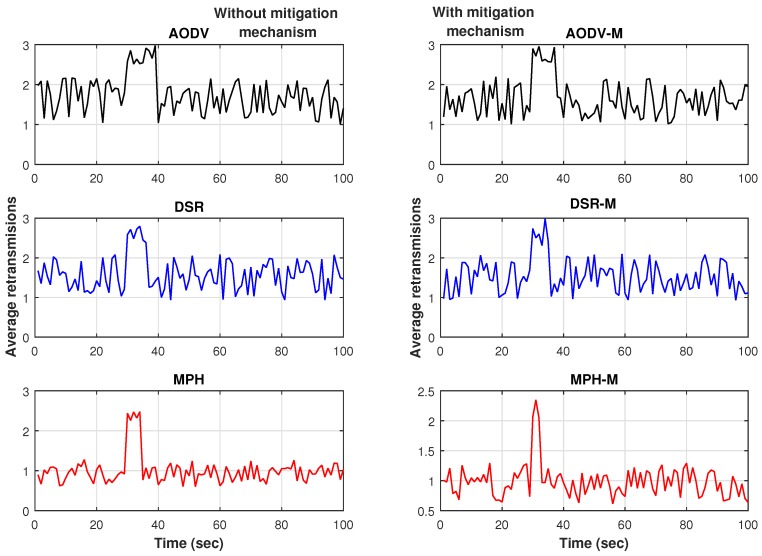
Resilience results for AODV, DSR and MPH and for AODV-M, DSR-M and MPH-M.

Figure 19 present the results for the resilience of the network, measured with a time-series produced by a variable associated to the packet retransmission average. The simulation time is 100 s. The jammer node is in the middle of the topology and the jammer attack starts at second 30. We can observe that MPH-M has around 15% lower exchange rate than AODV-M and DSR-M. In addition, the recovery time of MPH-M is 5 s faster than AODV-M and 2 s faster than DSR-M.

Table 9 shows the numerical values in seconds of the resilience of the network under each of the protocols studied. We observed that the mechanism of mitigation allows the reconfiguration of the links in the nodes be done in a faster way in each protocol.

## 7. Conclusions

In this paper, we have proposed a new mechanism for mitigation of jamming attacks, which is comprised of two phases: firstly the detection of the attack, and secondly the reaction of the network for adaptation of the routes avoiding those nodes nearby to the attack. For the detection, the method relies on an anomaly model, we have shown that the chosen variables of the model (CSMA retries, retransmissions, and energy) are suitable for building a jamming detector, where the metrics are combined under a majority voting rule, however some differences arise when the routing protocols are contrasted, MPH-M reaches higher efficiency in detection than DSR-M and AODV-M. The detection capabilities of the proposed method for MPH-M may stem from the behavior of the metrics, which jointly deliver a higher reliable decision since the deviations from the normal conditions are larger for the MPH-M protocol under jamming when compared to DSR-M and AODV-M.

The analysis carried out in the rest of the paper show that in general MPH-M receives more benefits from the mitigation scheme than DSR-M and AODV-M. For instance, the node energy for MPH-M is 138.13% better than AODV-M and 126.07% better than DSR-M. The other metrics considered in this work also show a remarkably higher performance for MPH-M: delay end-to-end, overhead and retransmissions. For CSMA retries the differences are lower among the three protocols, but again MPH-M has a better performance.

Furthermore, if we compare the results of the modified protocols with the performance of the original versions, we can observe that in general there is an increase in the performance, thus the network presents a mitigation of the jamming attack when the proposed method is integrated.

In this work, we compare the performance of our routing protocol, MPH, with two protocols widely known in the literature of WSNs: AODV and DSR. We made some modifications to these protocols in order to get a better detection mechanism. However, at present, there are other protocols in sensor networks, such as Routing Protocol for Low Power and Lossy Networks [30] and the modification of OSLR (Optimized Link State Routing) protocol made by [31], that we are going to work in the near future.

RPL is a proactive distance-vector routing protocol. This protocol is energy efficient in multipoint-to-point traffic scenarios, however, it presents inefficient performance in point-to-multipoint and point-to-point communications. Its main objective is the wireless networks of many nodes with limited resources and is managed by one or a few node collectors. It will be interesting, as a future work, to compare our MPH protocol with RPL.

In the modification of OSLR, the authors already made a performance analysis, but it is different to the performance analysis we made; therefore, it is not easy to compare. For example, the number of nodes is different, in our work we use 49 and they use 20; they use different topologies and our work is based on grid topology; they test with deceptive and random jamming and we use reactive jamming; they parametrize the jamming length, period and starting time and ours is fix; they limit their study mainly to MAC layer and we extend our study to MAC and Routing layer; the authors analyze the network performance in terms of communication and energy degradation and we use the energy consumption per node and the network as a whole, and we use more performance metrics such as retransmissions, overhead, affected nodes, and so on. Finally, we stress the network by increasing the traffic rate and the noise in the environment.

## Figures and Tables

**Table 1:** Simulation and real network parameters. CSMA/CA, carrier sense multiple access with collision avoidance [2].

Parameter	Value
**Physical Layer Parameters**	
Sensitivity threshold receiver	−94 dBm
Transmission power	4.5 dBm
Propagation model	Free Space
**MAC Layer Parameters**	
Waiting time for ACK packet	30 ms
Maximum retransmission number	3
Maximum retry number	5
Maximum number of tries to reach a node from the collector	9
Packet error rate	1%
Average frame length	22 bytes
Maximum number of backoffs	4
MAC protocol	IEEE 802.15.4
MAC layer	CSMA/CA
**Network Layer Parameters**	
Number of nodes	49
Maximum number of neighbors	16
Discovery neighbor time	30 s
Update time neighbors table	30 ms
Maximum data rate	250 kbps
Routing	Hierarchical
Scenario	Static nodes

**Table 2:** Nodes with positive detection of jamming.

Time (s)	Nodes in AODV
10	2-9-10-16-17-18-23-24-25-30-31-32
20	2-9-10-16-17-18-23-24-25-30-31-32
30	2-9-16-17-18-23-24-25-30-31-32
40	2-8-9-10-16-17-18-23-24-25-30-31-32-33
50	2-8-9-10-16-17-18-23-24-25-30-31-32
60	2-9-10-16-17-18-23-24-25-30-31-32
70	2-8-9-10-16-17-18-23-24-25-30-31-32
80	2-8-9-10-16-17-18-23-24-25-30-31-32
90	2-8-9-10-16-17-18-23-24-25-30-31-32-33
100	2-9-10-16-17-18-23-24-25-30-31-32
**Time (s)**	**Nodes in DSR**
10	2-9-10-16-17-18-23-24-25-30-31-32
20	2-9-10-16-17-18-23-24-25-30-31-32
30	2-8-9-16-17-18-23-24-25-30-31-32
40	2-8-9-10-16-17-18-23-24-25-30-31-32-33
50	2-8-9-10-16-17-18-23-24-25-30-31-32
60	2-9-10-16-17-18-23-24-25-30-31-32
70	2-8-9-10-16-17-18-23-24-25-30-31-32
80	2-9-10-16-17-18-23-24-25-30-31-32
90	2-8-9-10-16-17-18-23-24-25-30-31-32
100	2-8-9-10-16-17-18-23-24-25-30-31-32
**Time (s)**	**Nodes in MPH**
10	2-8-9-10-16-17-18-23-24-25-30-31-32
20	2-8-16-17-18-23-24-25-30-31-32
30	2-9-16-17-18-23-24-25-30-31-32
40	16-17-18-23-24-25-30-31-32
50	16-17-18-23-24-25-30-31-32
60	16-17-18-23-24-25-30-31-32
70	16-17-18-23-24-25-30-31-32
80	16-17-18-23-24-25-30-31-32
90	16-17-18-23-24-25-30-31-32
100	16-17-18-23-24-25-30-31-32

**Table 3:** Results of the jamming detector for the nodes under attack.

Node	Near			Node	Middle			Node	Far		
	**AODV**	**DSR**	**MPH**		**AODV**	**DSR**	**MPH**		**AODV**	**DSR**	**MPH**
0	✓	✓	✓	16	✓	✓	✓	40	✓	✓	✓
1	×	×	✓	17	✓	✓	✓	41	✓	✓	✓
2	✓	✓	✓	18	✓	✓	✓	47	×	✓	✓
7	×	✓	✓	23	✓	✓	✓	48	✓	✓	✓
8	✓	✓	✓	24	✓	✓	✓	-			
9	×	✓	✓	25	×	×	✓	-	-	-	-
14	✓	✓	✓	30	×	✓	✓	-	-	-	-
15	×	✓	×	31	✓	✓	✓	-	-	-	-
16	×	✓	✓	32	✓	✓	✓	-	-	-	-

**Table 4:** Retransmission Packets.

Packet Traffic Rate	% of Retransmission Packets
1	2.67
5	2.43
8	3.04
10	3.10
15	3.32
20	3.15
50	3.91
80	5.07
100	5.77
120	10.87

**Table 5:** Performance metrics per node under an uniform random traffic rate generation.

	Re-Transmissions	CSMA Retries	Overhead (%)	Delay End-to-End (s)	Energy (J)
**Without Mitigation Mechanism**					
**AODV**	2.51	4.06	60	1.256794	6.51
**DSR**	2.48	3.93	54	1.198735	6.23
**MPH**	2.21	3.60	38	0.716355	4.11
**With Mitigation Mechanism**					
**AODV-M**	2.28	3.91	51	1.209485	6.30
**DSR-M**	2.20	3.77	42	1.168756	6.12
**MPH-M**	1.98	3.39	27	0.589765	3.95

**Table 6:** Performance metrics per node under 10 packets/s traffic generation.

Without Mitigation Mechanism					
	**Re-Transmissions**	**CSMA Retries**	**Overhead (%)**	**Delay End-to-End (s)**	**Energy (J)**
**AODV**	2.41	3.44	63.5	1.385746	6.37
**DSR**	2.32	3.33	59.2	1.247636	6.02
**MPH**	1.93	2.75	41.7	0.816416	3.93
**With Mitigation Mechanism**					
**AODV-M**	1.92	2.33	48.7	1.088996	6.12
**DSR-M**	1.90	2.03	45.2	1.047822	5.81
**MPH-M**	1.11	1.91	27.3	0.516552	2.57

**Table 7:** A demonstration to show, for each metric, how better MPH is more efficient than AODV and DSR.

Without Mitigation Mechanism					
	**Re-Transmissions**	**CSMA Retries**	**Overhead**	**Delay End-to-End**	**Energy**
**AODV**	24.87%	25.09%	52.28%	69.74%	62.09%
**DSR**	20.21%	21.09%	41.97%	51.88%	53.18%
**With Mitigation Mechanism**					
**AODV-M**	72.97%	21.99%	78.39%	110.82%	138.13%
**DSR-M**	71.17%	6.28%	65.57%	102.85%	126.07%

**Table 8:** A demonstration to show, for each metric, how each protocol improves each metric when the mitigation scheme is used.

	Re-Transmissions	CSMA Retries	Overhead	Delay End-to-End	Energy
**AODV-M**	20.33%	32.27%	23.31%	21.41%	3.92%
**DSR-M**	18.10%	39.04%	23.65%	15.50%	3.49%
**MPH-M**	42.49%	30.55%	34.53%	36.73%	34.61%

**Table 9:** Resilience in seconds for AODV, DSR and MPH.

Protocol	Without Mitigation	With Mitigation
**AODV-M**	9 s	7 s
**DSR-M**	6 s	5 s
**MPH-M**	4 s	2 s
